# Development of a 2 μm Solid-State Laser for Lidar in the Past Decade

**DOI:** 10.3390/s23167024

**Published:** 2023-08-08

**Authors:** Kuan Li, Chao Niu, Chunting Wu, Yongji Yu, Yao Ma

**Affiliations:** Jilin Key Laboratory of Solid-State Laser Technology and Application, Changchun University of Science and Technology, Changchun 130022, China; 2022100002@mails.cust.edu.cn (K.L.); leoniuc@163.com (C.N.); yuyongjiyuyongji@163.com (Y.Y.); mayao@cust.edu.cn (Y.M.)

**Keywords:** 2 μm laser, all-solid-state laser, single longitudinal mode, lidar

## Abstract

The 2 μm wavelength belongs to the eye-safe band and has a wide range of applications in the fields of lidar, biomedicine, and materials processing. With the rapid development of military, wind power, sensing, and other industries, new requirements for 2 μm solid-state laser light sources have emerged, especially in the field of lidar. This paper focuses on the research progress of 2 μm solid-state lasers for lidar over the past decade. The technology and performance of 2 μm pulsed single longitudinal mode solid-state lasers, 2 μm seed solid-state lasers, and 2 μm high power solid-state lasers are, respectively, summarized and analyzed. This paper also introduces the properties of gain media commonly used in the 2 μm band, the construction method of new bonded crystals, and the fabrication method of saturable absorbers. Finally, the future prospects of 2 μm solid-state lasers for lidar are presented.

## 1. Introduction

The 2 µm band is eye-safe and corresponds to the absorption peaks of many gas and water molecules. Therefore, it has great value in the fields of lidar, space communication, biomedicine, gas detection, and material processing [1,2,3,4,5,6,7]. Moreover, the 2 µm band can be used as the pump source of the optical parametric oscillator to achieve mid-infrared laser output (3~5 µm, 8~12 µm), which is beneficial for military infrared countermeasures, laser guidance, laser spectroscopy, and other fields [8,9,10,11,12]. In biomedicine, the water absorption coefficient of the 2 µm laser is 600 cm^−1^, which is six orders of magnitude higher than that of visible light [13]. Therefore, the 2 µm laser is capable of achieving shallow biological tissue penetration depth and good thermal coagulation hemostasis in surgery, and can also be used for human tissue ablation, lithotripsy, and cutting when the water content is high [14,15,16,17,18]. In material processing, the 2 µm laser is in the eye-safe region, so it has little effect on the retina, with high sensitivity, which supports the full application of the 2 µm laser in industrial environments such as welding, cutting, printing, and film etching [19,20,21,22]. Among them, the most important is its application in lidar. The 2 µm laser is located in the atmospheric window with a low atmospheric absorption rate and can be transmitted to the middle and upper layers of the atmosphere (3~20 km). Meanwhile, CO_2_, N_2_O, and other gases also have absorption peaks in the 2 µm band, making this band suitable for gas detection, wind speed measurement, target recognition, and so on.

**Figure 1:**
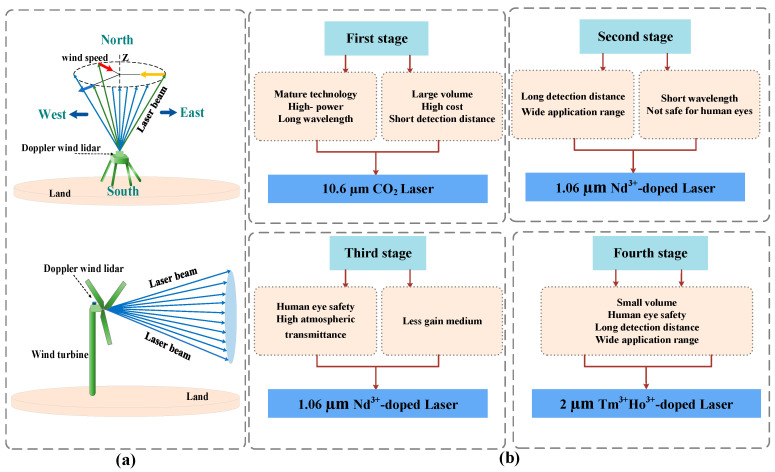
(**a**) Schematic diagram of lidar; (**b**) Schematic diagram of four stages of development of coherent Doppler wind lidar light source.

The coherent Doppler wind lidar for atmospheric wind field measurement is a current research hotspot. According to the wavelength of the laser emission light source, it can be divided into four categories. They are 10.6 µm CO_2_ laser [23,24], 1.06 µm Nd^3+^ doped solid-state laser [25,26,27], 1.55–1.6 µm Er^3+^ doped solid-state laser [28,29,30,31], and 2 µm Tm^3+^, Ho^3+^ doped solid-state laser. As shown in Figure 1, the detection principle of lidar and the development process of the four stages and their respective advantages and disadvantages are shown. According to the research [32,33], with the emergence of the fourth type of laser source, 2 µm Tm^3+^, Ho^3+^ doped solid-state laser, it not only overcomes the shortcomings of large volume, short detection distance, and less gain medium, but also greatly broadens the application range of lidar. In particular, the single-doped Ho^3+^ laser has a low quantum loss, small upconversion effect, insensitivity to operating temperature, and can obtain high power output, which meets the practical application requirements of lidar. At present, the 2 µm band solid-state laser has become the preferred light source for coherent Doppler wind lidar.

As the light source of coherent Doppler wind lidar, it not only strives for small size and low cost, but also meets its performance requirements. Coherent Doppler wind lidar measures the atmospheric wind field based on the coherent principle of laser, so the performance of the light source often has a decisive influence on the quality of lidar. Before 2010 [34,35], the research focus of the 2 µm solid-state lidar light source was mainly on the method of realizing the output of the 2 µm laser, such as looking for YAG, YAP, and other matrices with doped ions as the gain medium, and through the semiconductor laser or Tm^3+^ doped solid-state laser as the pump source, the continuous or pulse output of the 2 µm laser was realized. Although this kind of laser has the advantages of simple structure and high output power, it also has the disadvantages of poor beam quality, low repetition frequency, and narrow wavelength tuning range. In the past decade, the wind power industry has developed rapidly, and so has the demand for a 2 µm coherent Doppler wind lidar light source. This has shifted the research trend to improving the performance of the 2 µm laser. For example, research teams from the Beijing Institute of Technology and Harbin Institute of Technology use a non-planar ring oscillator (NPRO) or twisted-mode cavity method to eliminate the spatial hole burning effect and improve the output characteristics of 2 µm seed light with a single longitudinal mode, narrow linewidth, and high-frequency stability. On the other hand, research groups from Changchun University of Science and Technology, Shandong University, and Shandong Normal University use electro-optic modulation, acoustic-optic modulation, and new saturable absorber materials to control the loss of the resonant cavity, thereby improving the output power of the laser, optimizing pulse parameters and beam quality, and broadening the wavelength range and tuning ability. They aim to achieve as high as possible single pulse energy and as wide as possible pulse width so that coherent Doppler laser wind radar can have farther detection capability and higher measurement accuracy. In addition, in recent years, the performance of the 2 µm lidar light source has been enhanced. In addition to the continuous breakthroughs in laser technology, it has also benefited from the rapid development of crystal growth technology and coating technology, which has steadily improved the overall performance of the laser.

At the same time, a single longitudinal mode laser can provide highly accurate frequency control and stability, which is crucial for achieving accurate wind speed measurement. In addition, single-longitudinal-mode lasers with narrow linewidth can also provide a better range resolution and signal-to-noise ratio. The high-power laser can make the laser beam stronger, which makes the scattered signal easier to receive and analyze. It can also improve the penetration ability of the laser beam, so that the laser beam can pass through a longer distance, thereby expanding the measurement range of the laser radar. Therefore, single longitudinal mode and high power are usually regarded as the key elements of coherent Doppler wind lidar. Therefore, this paper will make a detailed introduction to the development process of a 2 µm solid laser light source suitable for coherent Doppler wind lidar in the past decade. The research status of the 2 µm pulse single longitudinal mode solid-state laser, 2 µm seed solid-state laser, and 2 µm high power solid-state laser is reviewed, and the development of the new crystal growth technology and the preparation technology of saturable absorber are introduced. Finally, the development of a 2 µm coherent Doppler wind lidar is prospected, hoping to provide some reference for readers.

## 2. Research Status of 2 µm Solid-State Lasers

In the research field of all-solid-state lasers, 2 µm band lasers have attracted much attention due to their characteristics of high conversion efficiency, compact structure, small size, and good stability. The common method to obtain a 2 µm laser output is generally an optical parametric oscillator using nonlinear frequency conversion technology, or directly pumping single-doped Tm^3+^, Ho^3+^ and Tm^3+^, Ho^3+^ co-doped gain medium. Due to the low conversion efficiency and complex structure of the former, and the output line width being wider when there is no single-frequency seed light injection, especially when the high-energy pulse output is more significant, the scheme of directly pumping doped Tm^3+^ and Ho^3+^ gain medium is more preferred in practical applications. Therefore, at present, single-doped Tm^3+^ solid-state laser, single-doped Ho^3+^ solid-state laser, and Tm^3+^, Ho^3+^ double-doped solid-state laser are the main technical ways to obtain 2 µm band laser output. Multi-faceted research has been carried out on them, including the preparation of new laser crystals, and devices and exploration of new technical methods.

### 2.1. Research Status of 2 µm Pulsed Single Longitudinal Mode Solid-State Lasers

As shown in Table 1, injection locking technology has become the preferred technology for achieving single-longitudinal-mode high-energy pulsed lasers output with 2 µm solid-state lasers. This technology involves injecting a continuous single longitudinal mode seed laser with narrow linewidth and low power into a high-power laser oscillator and using the Ramp–Hold–Fire, Ram-pFire, and Pound–Drever–Hall technology to achieve successful injection locking. By inserting a Q-switched device into the cavity to amplify the seed laser multiple times, the high-energy single-frequency laser output with pulse energy up to mJ and repetition frequency up to kHz is finally achieved. In the past decade, relevant research in this field has been carried out by representative teams at home and abroad. In 2016, a 2 µm single-frequency pulsed laser with Ho: YAG ceramics was achieved by injection locking technology by the Beijing Institute of Technology, and a single pulse energy of 14.76 mJ was obtained. In order to further improve the pulse energy of a single-frequency 2 µm laser, the seed injection locking composite master oscillator and power amplifier (MOPA) technology was adopted for the first time in 2017, and the two-stage amplification was adopted. The first-stage amplifier adopted a double-end pumping configuration, and the second-stage amplifier adopted a single-end pumping configuration, which further improved the energy of single-frequency pulses and achieved a high pulse energy of 55.64 mJ. This is the largest pulse energy known to Ho: YAG lasers, which is of great help for realizing remote measurement.

**Table 1:** 2 µm pulsed single longitudinal mode solid-state laser.

Reference	Year	Research Establishment	Output Wavelength (nm)	Method	Laser Substance	Repetition Frequency (Hz)	Pulse Width (ns)	Output Energy (mJ)	Line Width (MHz)
[36]	2010	Beijing Institute of Technology	2 µm	Injection seed	Tm: YAG	200	290	2.23	2
[37]	2016		2090	Injection seed	Ho: YAG	200	109	15.15	4.19
[38]	2016		2090	Injection seed	Ho: YAG Ceramics	200	121.6	14.76	3.84
[39]	2016		2090	Injection seed	Ho: YAG	250	122	17.04	3.82
[40]	2017		2090	Injection seed	Ho: YAG	1000	172	6.24	2.61
[41]	2017		2090	Injection seed	Ho: YAG	252	220	8.8	2.48
[42]	2017		2090	Injection seed MOPA	Ho: YAG	150–750	102–215	31.4–12.7	-
[43]	2017		2090	Injection seed MOPA	Ho: YAG Ceramics	200	121	55.64	3.96
[44]	2018		2090	Injection seed MOPA	Ho: YAG	1250	178.9	13.76	2.65
[45]	2012	Harbin Institute of Technology	2090.9	Injection seed	Ho: YAG	100	132	7.6	3.5
[46]	2012		2090	Injection seed	Ho: YAG	110	110	11	4.8
[47]	2013		2118	Injection seed	Ho: YAIO_3_	100	151	8	3.7
[48]	2018		2050.967	Injection seed	Ho: YLF	100	65	4.4	4.1 (132 ns)
[49]	2014	Ecole Polytechnique	2 µm	Injection seed	Ho: YLF	2000	42	13.5	11
[50]	2015		2 µm	Injection seed	Ho: YLF	2000	40	10	10
[51]	2018		2051.01 2051.25	Injection seed MOPA	Ho: YLF	303.5	33 74	12 42	6.1 14.0
[52]	2011	Council for Scientific and Industrial Research, South Africa	2064	Injection seed MOPA	Ho: YLF Ho: LuLF	50	350	210	-
[53]	2013		2064	Injection seed MOPA	Ho: YLF	50	350	325	-
[54]	2016	Fraunhofer Insitute for Laser Technology	2051	Injection seed	Ho: YLF	1000 100	10	2.7 6.7	-
[55]	2020	Université Paris Saclay	2 µm	Volume Bragg Gratings YAG etalon	Tm: YAP	1000	50	0.230	-

**Figure 2:**
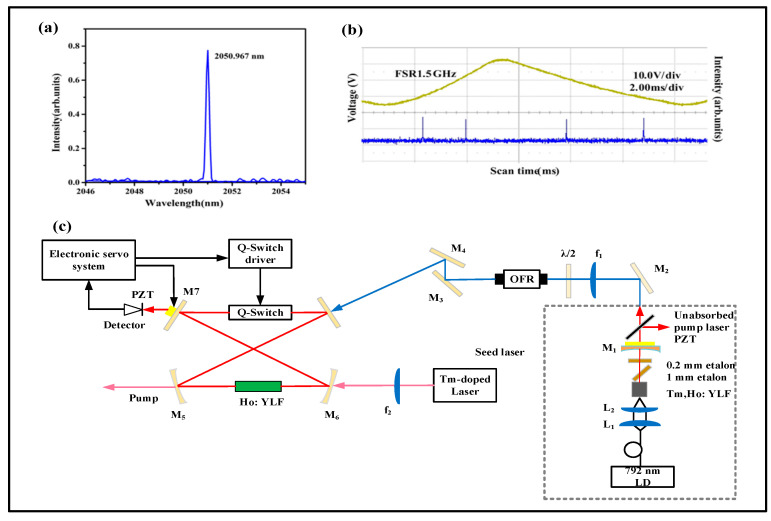
(**a**) Wavelength of Tm, Ho: YLF seed laser; (**b**) F-P scanning spectrum of single-frequency Tm, Ho: YLF laser; (**c**) Experimental setup of seed-injected Ho: YLF laser Reprinted with permission from [48] © Elsevier.

In 2018, the Harbin Institute of Technology studied a tunable single longitudinal mode injection seeded Q-switched Ho: YFF laser, as shown in Figure 2. Firstly, two Fabry–Perot etalons were inserted into the Tm, Ho: YLF laser resonator and the cavity length was changed by controlling the piezoelectric transducer. A tunable single longitudinal mode seed laser with a wavelength of 2050.962 nm~2051.000 nm was realized. Secondly, the single-frequency seed laser was injected into the Q-switched Ho: YLF laser by using the ramp–hold–fire technique. The pulse output energy was 4.4 mJ and the pulse width was 65 ns at a repetition rate of 100 Hz.

**Figure 3:**
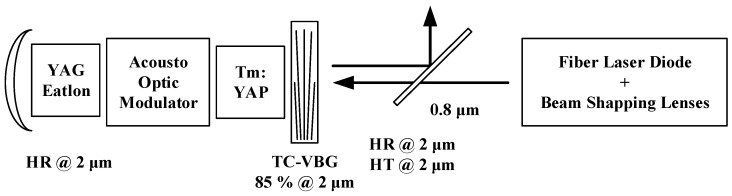
2 µm Tm: YAP single longitudinal mode laser [55].

Compared with the domestic injection locking technology, 2 µm pulsed single longitudinal mode laser output was achieved by foreign research institutions by directly inserting Q-switched elements into the resonant cavity. This method has a simpler structure, higher stability, smaller volume, and easier adjustment. In 2020, single longitudinal mode operation was realized by Université Paris Saclay by using volume Bragg grating and etalon in a straight cavity structure, as shown in Figure 3. At the same time, a single longitudinal mode laser output with a single pulse energy of 230 µJ and a pulse width of 50 ns at a repetition rate of 1 kHz was achieved by inserting an acoustic-optic modulator into the cavity.

In addition, in terms of realizing pulsed single longitudinal mode, the gain medium with single doping of Ho^3+^, mainly consisting of Ho: YAG and Ho: YLF, is selected as the laser material at home and abroad, according to the crystal parameters in the above table. The reason is that they have a large emission cross section (10^−20^ cm^−2^) and a long upper-level lifetime (10 ms), and the Ho^3+^ ion has a small quantum loss and less heat generation during the transition from ^5^I_7_ to ^5^I_8_ level to 2 µm, which is beneficial to improving the conversion efficiency of the laser. At the same time, compared with Tm^3+^ and Ho^3+^ co-doping, the single doping of Ho^3+^ can reduce the conversion loss of the upper level, and it is easier to realize the single-frequency laser output with a high repetition rate and large pulse energy. On the other hand, due to the complexity of realizing 2 µm pulse single longitudinal mode technology, in recent years, by optimizing the coating design and improving the coating process, such as using a multi-objective optimization algorithm, physical model-based design method, machine learning-based design method [56,57], etc., the coating performance can be accurately predicted and optimized to meet the complex needs of multi-band, broadband, and large angle. For example, advanced coating processes such as ion beam sputtering, atomic layer deposition, and chemical vapor deposition are used. These technologies can achieve precise control of the thickness of the coating layer, improve the uniformity and compactness of the coating structure, and reduce the stress of the coating. They have made a great contribution to the realization of the high repetition frequency and high energy pulse light.

Therefore, combined with the development of 2 µm pulsed single longitudinal mode lasers in the past decade, the future will continue to be dominated by injection-locking technology, and higher pulse energy will be achieved by multi-stage amplification through injection-locking composite MOPA technology. In terms of materials, a new gain medium based on single-doped Ho^3+^ will also be continued to be developed, and the doping concentration and length of the gain medium will be optimized to improve the conversion efficiency, reduce the thermal effect, and achieve high-power pulsed light output.

### 2.2. Research Status of 2 µm Seed Solid-State Laser

According to the single longitudinal mode of 2 µm pulse, the laser Doppler wind radar requires its seed laser to have continuous single longitudinal mode output, stable frequency, and narrow linewidth.

**Figure 4:**
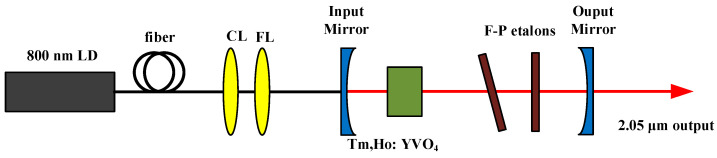
Double Fabry–Perot etalons Tm, Ho: YVO_4_ laser experimental setup. Reprinted with permission from [65] © Elsevier.

As shown in Table 2, the methods of obtaining a single longitudinal mode mainly include the Fabry–Perot etalon method, microchip laser method, twisted-mode cavity method, nonplanar ring oscillator method, coupled-cavity method, etc. Among many methods, the method of inserting one or more Fabry–Perot etalons directly into the cavity and changing the angle of the etalon to obtain tunable narrow linewidth single longitudinal mode laser output is the most commonly used method because of its relatively easy operation. In 2015, the Harbin Institute of Technology realized single longitudinal mode output by the microchip laser method and double Fabry–Perot structure, as shown in Figure 4. In the case of using the microchip laser method, the single longitudinal mode output power of 17 mW is obtained when the wavelength is 2052.4 nm. In order to further improve the output power, the double F-P etalon is placed in the cavity of a Tm, Ho: YVO_4_ laser. With the change of the angle of the F-P etalon, the wavelength of the single longitudinal mode laser can change from 2050.4 nm to 2051.3 nm, and the output power is as high as 95 mW at the wavelength of 2051.3 nm. However, Fabry–Perot etalon itself is a high-loss device. When the pumping power is high, there will be multiple longitudinal mode outputs, which will lead to the failure to achieve a big breakthrough in optical–optical conversion efficiency and a small wavelength tuning range. Therefore, it is necessary to accurately control the temperature of the etalon in the experiment.

**Table 2:** 2 µm single longitudinal mode seed laser.

Reference	Year	Research Establishment	Method	Output Wavelength (nm)	Pump Source Wavelength (nm)	Laser Substance	Output Power (mW)
[58]	2010	Harbin Institute of Technology	Microchip Laser	2039.7598	802 nm LD	Tm, Ho: GdVO_4_	26.4
[59]	2011		Microchip Laser	2050.5	785 nm LD	Tm, Ho: YLF	17
[60]	2011		Microchip Laser	2102.6	791.7 nm LD	Tm, Ho: YAP	42
[61]	2011		0.1 mm F-P 1 mm F-P	2013.91	792 nm LD	Tm: YAG	60
[62]	2012		0.05 mm F-P 1 mm F-P	1897.6	792 nm LD	Tm: GdVO_4_	34
[63]	2012		0.05 mm F-P 1 mm F-P	2118.09	792 nm LD	Tm, Ho: YAP	57
[64]	2013		0.5 mm F-P 1 mm F-P	2015.87	785 nm LD	Tm: YAG Ceramic	318
[65]	2015		Microchip Laser 0.5 mm F-P 0.05 mm F-P	2052.4 2051.3	800 nm LD	Tm, Ho: YVO_4_	17 95
[66]	2016		0.1 mm F-P 1 mm F-P 6 mm F-P	2081.2	1908 nm Tm: YLF laser	Ho: YAG	309
[67]	2016		1 mm F-P 6 mm F-P	2051.6	1.94 µm Tm-doped fiber laser	Ho: YLF	345
[68]	2017		Unidirectional ring laser	2053.9	1938 nm Tm: YAP laser	Ho: YVO_4_	941
[69]	2017		Unidirectional ring laser	2063.8	1938 nm Tm-doped laser	Ho: YLF	3.73 × 10^3^
[70]	2017		Unidirectional ring laser	2051.65	1.94µm Tm-doped fiber laser	Ho: YLF	528
[71]	2014	Harbin Engineering University	0.1 mm F-P 1 mm F-P	2064.4	792 nm LD	Tm, Ho: LLF	221
[72]	2017		1 mm F-P 6 mm F-P	2111.91	1907 nm Tm: YAP laser	Ho: Sc_2_SiO_5_	590
[73]	2011	Beijing Institute of Technology	Coupled-cavity	1990	793 nm LD	Tm: YAP	784
[74]	2012		Twisted-mode cavity	2 µm	785 nm LD	Tm: YAG	1.46 × 10^3^
[75]	2013		Nonplanar ring oscillator	2122.213	1907 nm Tm: YLF laser	Ho: YAG	8.0 × 10^3^
[76]	2013		Nonplanar ring oscillator	2091	1907 nm Tm: YLF laser	Ho: YAG	3.09 × 10^3^
[77]	2018	Luoyang Institute of Electro-optical Equipment	0.1 mm F-P 1 mm F-P	2022.64	785 nm LD	Tm: LuAG	93
[78]	2019	Nanjing University of Information Science and Technology	Unidirectional ring laser	2055.86	792 nm LD	Tm, Ho: YAP	231

In addition, the coupled-cavity method is also an efficient method to obtain single longitudinal mode output. In 2011, a compact single longitudinal mode Tm: YAP laser was studied by using the coupled cavity method at the Beijing Institute of Technology. The resonant cavity is formed by a planar input mirror and the side of the laser crystal M2. The other resonant cavity is composed of the side of the laser crystal M2 and a concave output mirror. The resonant frequency of the two cavities can be oscillated at the same time. No mode selection element is used in the cavity, and the single longitudinal mode oscillation is achieved by coupling two cavities. A single-frequency output power of 784 mW is obtained at 1990 nm, and the slope efficiency is 52%. However, in the experiment, it is difficult to adjust the coupled cavity, and the length of the laser medium suitable for the coupling cavity method is generally not more than 1.5 mm, so there is a phenomenon of small output power.

**Figure 5:**
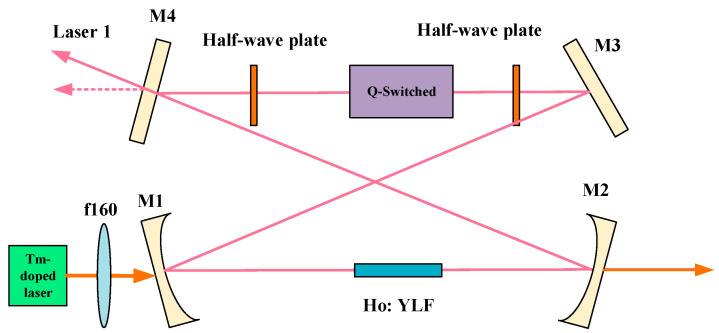
Single longitudinal mode Ho: YLF unidirectional ring laser. Reprinted with permission from [69] © Elsevier.

In recent years, by inserting an acoustic-optic modulator, a Faraday rotator, and a half-wave plate into a unidirectionally operating ring cavity to change the loss of laser in different directions in the cavity, a single longitudinal mode laser with high output power can be obtained. In 2017, the Harbin Institute of Technology designed a Ho: YLF single longitudinal mode laser by inserting an acoustic-optic modulator and two half-wave plates into a single ring cavity, as shown in Figure 5. When the incident pump power is 16.4 W, the single longitudinal mode output power can reach 3.73 W, and the corresponding slope efficiency is 27.1%.

In addition, including the twisted-mode cavity method and the nonplanar ring oscillator method, combined with different types of doped media, the single longitudinal mode seed laser with better parameters is also realized. However, as the light source of coherent Doppler wind lidar, it is necessary to amplify the seed laser to achieve a longer distance and more accurate measurement. Therefore, the research status of 2 µm high-power solid-state lasers will be reviewed.

### 2.3. Research Status of 2 µm High Power Solid-State Lasers

#### 2.3.1. 2 µm Actively Q-Switched Pulsed Laser

In order to meet the requirement that coherent Doppler wind lidar can have a longer detection distance, it cannot be achieved by seed laser alone. Therefore, the light source needs to have a sufficiently high single pulse energy and an adequately wide pulse width. Therefore, a 2 µm high-power solid-state laser is particularly important. This paper will introduce 2 µm high power solid-state lasers by two technical means, namely active modulation and passive modulation.

**Figure 6:**
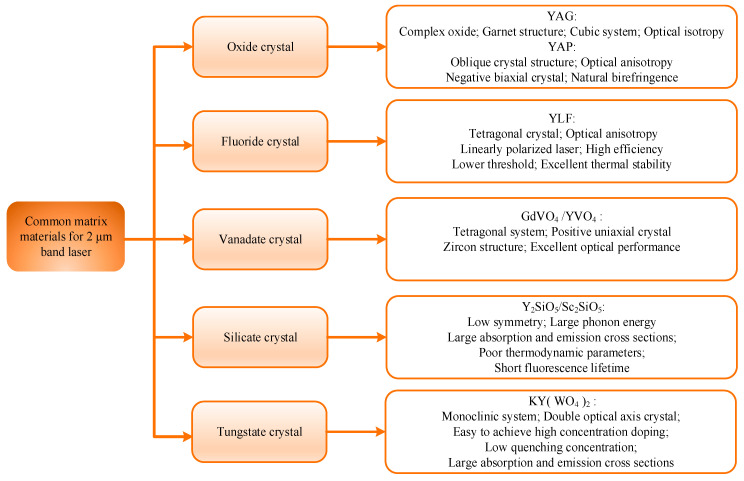
Common working substances in the 2 µm band.

According to Table 3, in order to realize the output of a 2 µm high-power laser, the relevant research team has made corresponding attempts in the crystal matrix, crystal structure, pumping mode, and Q-switching mode. First of all, due to the differences in the structural composition of the matrix material and the crystal field effect, the output performance also shows different results after doping rare earth ions. First of all, the materials of a cubic crystal system are generally more transparent than those of a tetragonal crystal system. When the laser passes through the crystal material, it is not easy to affect by absorption and scattering, and the cubic crystal system has a higher refractive index, which can produce higher output power, but also produce a strong loss. Secondly, the crystal field can control the optical properties of laser materials by adjusting the position and arrangement of ions or atoms [79,80]. For example, the crystal field can effectively adjust the refractive index, dispersion, absorption cross-section, and other parameters of the material, so as to achieve accurate control of the laser wavelength and output power. Therefore, it is very important for laser design to choose the right crystal material and crystal structure to further optimize the output characteristics of the laser. The most commonly used substrate materials for achieving laser output in the 2 µm band are oxides (YAG, YAP), fluorides (YLF), vanadate crystals (GdVO_4_), silicate crystals, and tungstate crystals (KYW), whose classification and properties are shown in Figure 6.

**Table 3:** 2 µm actively Q-switched solid-state laser.

Reference	Year	Research Establishment	Output Wavelength (nm)	Method	Laser Substance	Repetition Frequency (kHz)	Pulse Width (ns)	Output Energy (mJ)
[81]	2018	Changchun University of Science and Technology	2014.9	AO	3 × (3 + 8) mm^3^ Tm (3.5 at.%): YAG	200 × 10^−3^	367.7	6.83
[82]	2019		1.99 µm	AO	3 × 3 × 15 mm^3^ Tm (3 at.%): YAP	1	38.04	16.36
[83]	2020		1937.87	EO	2 × 6 × 15 mm^3^ Tm (2.5 at.%): YAP	10	20.64	2.20
[84]	2021		2014.16	AO	3 × (5 + 12 + 5) mm^3^ Tm (3.5 at.%): YAG	100 × 10^−3^	416 407	3.6
[85]	2021		2129.22	AO	4 × 4 × 25 mm^3^ Ho (0.8 at.%): YAP	10	104.2	-
[86]	2015	Shandong University	2 µm	AO graphene	4 × 4 × 8 mm^3^ Tm (6 at.%): LuAG	1	170	-
[87]	2017		2 µm	AO	Tm (3 at.%),Y(3 at.%):CaF_2_	1	280	0.335
[88]	2019		2118	EO	3 × 3 × 10 mm^3^ Ho (3 at.%): YAP	4	33	1
[89]	2019		2 µm	AO g-C_3_N_4_	3 × 3 × 10 mm^3^ Tm (3 at.%): YAP	-	239	-
[90]	2021		2 µm	EO VS_2_	3 × 3 × 10 mm^3^ Tm (3 at.%): YAP	200 × 10^−3^	22	755 × 10^−3^
[91]	2021		2 µm	EO Sb_2_Te_3_	3 × 3 × 10 mm^3^ Tm (3 at.%): YAP	100 × 10^−3^	38	-
[92]	2014	Harbin Institute of Technology	2013	AO	7 × 7 × 1.5 mm^3^ Tm (3.5 at.%): YAG	200	54	-
[93]	2014		1989	AO	3 × 3 × 6 mm^3^ Tm (3.5 at.%): YAP	200	43	-
[94]	2014		2048.2	AO	4 × 4 × 20 mm^3^ Ho (1.0 at.%): GdVO_4_	5	4.7	0.9
[95]	2015		1996.9	EO	1.5 × 6 × 40 mm^3^ Tm (0.15 at.%): YAP	100	7.1	-
[96]	2018		2090.7	AO	Ho (0.5 at.%): YAG	20	21	5.3
[97]	2012	University of Hamburg	2.09 µm	AO	Ho (0.5 at.%): YAG	100 × 10^−3^	100	30
[98]	2013	Beijing Institute of Technology	2097	AO	Ho: YAG ceramic	100 × 10^−3^	-	10.2
[99]	2015	RIKEN Center of Advanced Photonics	2.01 µm	AO	Tm (3 at.%): YAG	10 × 10^−3^	160	128
[100]	2016	Tsinghua University	2.06 µm	AO	Ho (0.5 at.%): YLF	100 × 10^−3^	43	1.1
[101]	2017	Heilongjiang Institute of Technology	2119	AO	4 × 4 × 10 mm^3^ Tm (0.5 at.%), Ho (0.5 at.%): YAP	7.5	-	-
[102]	2019	Prokhorov General Physics Institute of the Russian Academy of Sciences	2 µm	AO	4 × 4 × 4 mm^3^ Tm (5.7 at.%): YbAG	6.7	45	100 × 10^−3^
[103]	2020	Feng Chia University	1985-1940	EO	Tm (4 at.%): YAP	1	60	2
[104]	2021	Tianshui Normal University	1944	AO	1.5 × 6 × 30 mm^3^ Tm (2 at.%): YAP	40	64	-
[105]	2021	Institute of Physics of the Czech Academy of Sciences	1.88 µm	EO	Tm: YAG	1 × 10^−3^	18	2.22
[106]	2021	Changchun Institute of Technology	2.1 µm	EO	Ho: GdVO_4_	5	4.6	0.9
[107]	2021	Changchun Institute of Optics, Fine Mechanics and Physics	2.06 µm	AO	3 × 3 × 14 mm^3^ Tm (2 at.%): Lu_2_O_3_	5	46	0.74
[108]	2022	Jerusalem College of Technology	1940	EO	Tm: YAP	1	20	2.76

**Figure 7:**
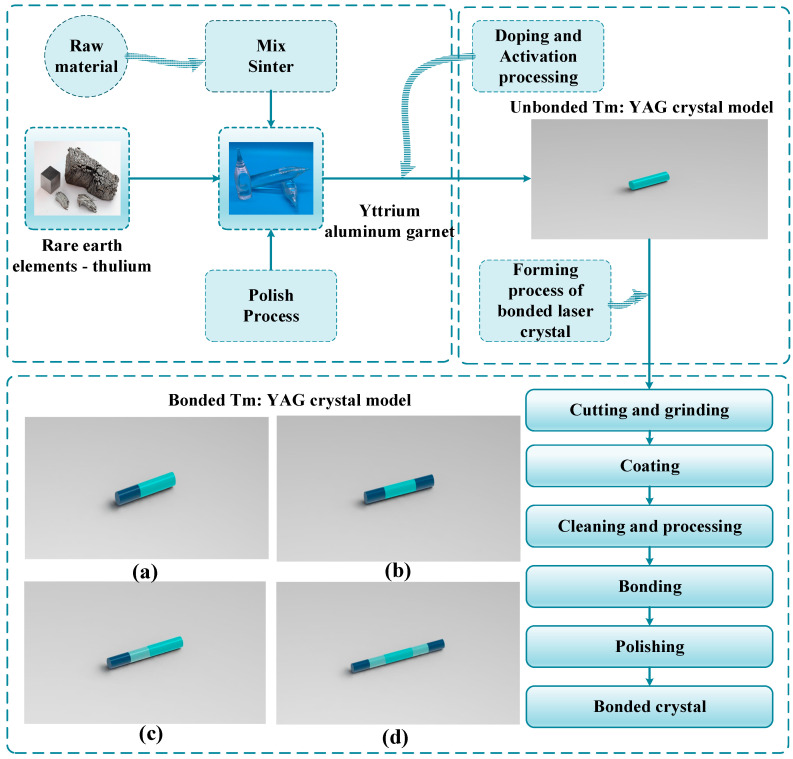
Common Tm: YAG bonded crystal model. (**a**) Tm: YAG single-ended bonded crystal model; (**b**) Tm: YAG double-ended bonded crystal model; (**c**) Tm: YAG concentration gradient crystal model; (**d**) Tm: YAG multi-segment bonded crystal model.

The above table also shows that the single doped Tm^3+^ crystals have become the gain medium of choice for obtaining high heavy frequency, high energy 2 µm bands, such as Tm: YAG, Tm: YAP, Tm: LuAG, due to the strong absorption peak near 790 nm of the Tm^3+^ doped gain medium, which can be directly selected for pumping by commercial LD with output wavelength in the range of 1.9 µm–2.1 µm. However, due to the small emission cross section and low gain of the single-doped Tm^3+^ laser, there is a certain limitation to the Q-switched operation to improve the single pulse energy, and the Tm^3+^ energy level structure is a quasi-triple energy level system, which will lead to the stability of the laser in high power operation, the thermal effect of the crystal, and in serious cases may cause damage to the crystal. Therefore, relevant research groups have adopted new structures of crystals such as bonded crystals and concentration gradient crystals to mitigate the effects of thermal effects. Bonded crystals are a series of methods such as mechanical processing, coating, pasting, heat treatment, and polishing of multiple single crystals to form a crystal with a higher damage resistance threshold and a wider operating temperature range, which can mitigate the effects of thermal stress on the crystal due to the presence of more structural defects and interfaces within it that can absorb and disperse heat. Concentration gradient crystals are crystals with non-uniformly distributed refractive indices that gradually change inside the crystal along one or more directions, allowing for maximum amplification at each position as the laser propagates through the crystal, and also reducing thermal effects by adjusting the concentration distribution inside the crystal. The preparation process of bonded crystals is shown in Figure 7a–d in the figure are common types of bonded Tm: YAG crystals model, respectively.

**Figure 8:**
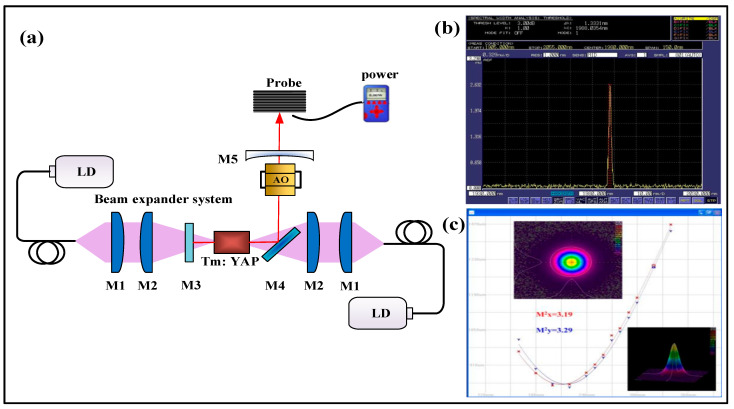
(**a**) Double-end-pumped high energy acoustic-optic Q-switched Tm: YAP laser; (**b**) Wavelength of Tm: YAP laser; (**c**) Beam quality of pulse Tm: YAP laser [82].

According to the research, the research groups represented by Changchun University of Science and Technology, the Harbin Institute of Technology, and Shandong University have realized the single pulse energy output of ns magnitude pulse width and mJ magnitude through 2 µm active Q-switched technology. In 2018, Changchun University of Science and Technology first proposed the use of an intermittently pumped 2 µm acoustic-optic Q-switched Tm: YAG laser with a crystal of 3 × (3 + 8) mm^3^ Tm: YAG bonded crystal. When the pump energy is 86.2 mJ and the repetition frequency is 200 Hz, the maximum output energy of 6.83 mJ and the minimum pulse width of 367.7 ns are obtained, and the laser stability is greater than 98%. The following year, the team studied the work of a double-ended pumped high-energy acoustic-optic Q-switched Tm: YAP laser. When the pump power is 79.2 W and the repetition frequency is 1 kHz, the laser output with a wavelength of 1.99 µm is obtained. The pulse energy is as high as 16.36 mJ, the pulse width is 38.04 ns and the peak power is 430.07 kW. The slope efficiency is 29.42% and the optical-to-optical conversion efficiency is 20.66%. The experimental device is shown in Figure 8. According to the realization method of the above table, the active Q-switched technology is divided into two technical means: electric-optic modulation and acousto-optic modulation. The electro-optic Q-switched technology has a strong turn-off ability, which can achieve high energy or narrow linewidth amplification. The repetition frequency is adjustable, and the output of high repetition frequency or low repetition frequency can be realized. However, it requires a high-voltage drive device, which increases the complexity and cost of the system and is sensitive to polarized light. It is necessary to place polarizers or use linearly polarized light. Therefore, in the past decade, many research groups have adopted acoustic-optic Q-switched technology. This technology has a simple structure, is suitable for low-gain lasers, and can obtain high-repetition-rate and high-energy pulse light. It is very suitable as a way to amplify the coherent Doppler wind lidar seed light source.

#### 2.3.2. 2 µm Passively Q-Switched Pulsed Laser

Passively Q-switched technology is to set a saturable absorber in the laser resonant cavity and uses its saturable absorption effect to periodically control the resonant cavity loss to obtain a pulsed light output. In the past decade, remarkable progress has been made in the preparation process and method of a saturable absorber with the breakthrough of a 2 µm saturable absorber in new two-dimensional nanomaterials, which has also been favored by many research groups.

**Figure 9:**
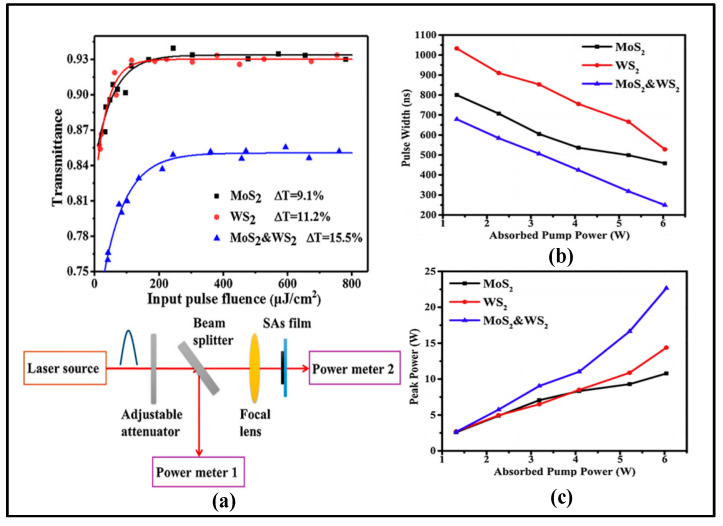
(**a**) Nonlinear transmittance of MoS_2_, WS_2_, and MoS_2_ & WS_2_ and the experimental setup of absorption measurement; (**b**) Variation of pulse width with pumping power for MoS_2_, WS_2_, and MoS_2_ & WS_2_; (**c**) Variation of peak power with pumping power for MoS_2_, WS_2_, and MoS_2_ & WS_2_. Reprinted with permission from [116] © Elsevier.

As shown in Table 4, the saturable absorber is a key component of passive Q-switching technology. In the past decade, many new saturable absorber materials have emerged, including graphene, black phosphorus, carbon nanotubes, transition metal diols, topological insulators, etc. These materials have a higher damage threshold, lower saturation absorption intensity, wider absorption bandwidth, and faster recovery time, which can achieve higher output power, narrower pulse width, and higher repetition frequency. The 2 µm passively Q-switched solid-state laser is mainly represented by Shandong University. In 2022, Shandong University obtained a stable pulse output by applying MXene VCrC nanosheets prepared by the liquid phase exfoliation method to 2 µm passively Q-switched Tm: YAP. When the pump power is 3.52 W and the repetition frequency is 49 kHz, the output power of 280 mW is achieved by using the output mirror with T = 3%. The pulse energy is 5.7 µJ and the peak power is 6.6 W. In order to further improve the performance of the laser, Shandong University has realized the dual passively Q-switched 2 µm laser output. In 2019, Shandong University simultaneously placed WS_2_ and MoS_2_ in the resonant cavity to realize a 2 µm dual passively Q-switched Tm: YAP laser. At a repetition rate of 62.7 kHz, a pulse width of 249.4 ns and a maximum peak power of 22.4 W was obtained. The experimental results are shown in Figure 9. The modulation depths of the single saturable absorber and two saturable absorbers shown in (a) are 9.1%, 11.2%, and 15.5%, respectively. The results show that the modulation depth of the double saturable absorbers is the largest. The modulation depth of the saturable absorber has an important influence on the Q-switched effect of the laser. The greater the modulation depth, the more the saturable absorber can absorb more photons, thus enhancing its nonlinear absorption characteristics and increasing the saturable light intensity of the material, which can effectively increase the loss of the laser and thus achieve a better modulation effect. Therefore, the modulation depth of the saturable absorber needs to be controlled when designing and preparing passive Q-switched elements to ensure the best performance and reliable operating characteristics.

**Table 4:** 2 µm passively Q-switched solid-state laser.

Reference	Year	Research Establishment	Output Wavelength (nm)	Saturable Absorber	Laser Substance	Repetition Frequency (kHz)	Pulse Width (ns)	Output Energy (µJ)
[109]	2014	Shandong University	2 µm	graphene	3 × 3 × 7 mm^3^ Tm (3 at.%): YAP	42.4	735	8.5
[110]	2016		2012.9	WS_2_	4 × 4 × 8 mm^3^ Tm (6 at.%): LuAG	63	-	-
[111]	2016		1969 1979	BP	3 × 3 × 7 mm^3^ Tm(3 at.%):YAP	81	181	39.5
[112]	2017		2023	Bi_2_Te_3_	4 × 4 × 8 mm^3^ Tm(6 at.%): LuAG	118	620	-
[113]	2018		1977	MoTe_2_	3 × 3 × 8 mm^3^ Tm(3 at.%):YAP	144	380	8.4
[114]	2018		2021.7	Bi_2_Te_3_	4 × 4 × 8 mm^3^ Tm: LuAG	-	233	-
[115]	2019		2 µm	WSe_2_	4 × 4 × 10 mm^3^ Tm(3 at.%): YLF	82	427	12.8
[116]	2019		2 µm	MoS_2_ WS_2_	3 × 3 × 5 mm^3^ Tm(1.0 at.%): YAP	62.7	249.4	-
[117]	2020		2015	Bi_2_Se_3_	3 × 3 × 6 mm^3^ Tm(5 at.%): YAG ceramic	148.5	355	6.76
[118]	2020		2 µm	SWCNTs	3 × 3 × 10 mm^3^ Tm (2 at.%): YLF	35.9	920	-
[119]	2020		2004	Mg-MOF-74	3 × 3 × 10 mm^3^ Tm (3 at.%):YAP	117	313	5.6
[120]	2022		2 µm	VCrC	Tm: YAP	49	658	5.7
[121]	2016	Shandong Normal University	1988	BP	3 × 3 × 4 mm^3^ Tm (5 at.%):YAP	19.25	1.78 × 10^3^	7.84
[122]	2018		2 µm	graphene	3 × 3 × 7 mm^3^ Tm (4 at.%), Y(4 at.%):CaF_2_	20.22	1.316 × 10^3^	20.4
[123]	2018		1935.4	Ag-NRs	3 × 3 × 7 mm^3^ Tm (4 at.%): CaF_2_	9.3	3.1 × 10^3^	41.4
[124]	2019		2 µm	Ti_3_C_2_T_x_	3 × 3 × 7 mm^3^ Tm (3 at.%), Gd (0.5 at.%): CaF_2_	19.61	2.39 × 10^3^	-
[125]	2021		1937.9	Mo: BiVO_4_	3 × 3 × 10 mm^3^ Tm (3 at.%): YAP	70.08	821	2.18
[126]	2021		1936.6	graphdiyne	3 × 3 × 8 mm^3^ Tm (3 at.%): YAP	98.59	-	13.85
[127]	2021	Qingdao University of Science & Technology	2 µm	NiV-LDH	3 × 3 × 6 mm^3^ Tm (6 at.%): YAG ceramic	101.8	398	2.30
[128]	2021		2 µm	NiCo-LDH	3 × 3 × 6 mm^3^ Tm (6 at.%): YAG ceramic	119.3	322.6	2.10
[129]	2019	Shandong University of Science and Technology	1937.8	ReSe_2_	Tm: YAP	89.4	925.8	17.6
[130]	2020		2 µm	Ta_2_NiSe_5_	3 × 3 × 8 mm^3^ Tm (3 at.%): YAP	71	740	6.35
[131]	2020		2 µm	Mo_0.5_Re_0.5_S_2_	Tm: YAP	95	857.5	10.1
[132]	2018	Fujian Institute of Research on the Structure of Matter	2012.6	Bi_2_Te_3_	3 × 3 × 4 mm^3^ Tm (3.5 at.%): YAG	57.67	382	4.8
[133]	2018		2 µm	MoS_2_	3 × 3 × 4 mm^3^ Tm (3.5 at.%): YAG	49.36	423	8.53
[134]	2016	Jiangsu Normal University	2 µm	Gold nanorods	3 × 3 × 10 mm^3^ Tm (3 at.%): YAG	77	796	-
[135]	2016	Universitat Rovira i Virgili	1929	MoS_2_	Tm (5 at.%): KLuW	170	175	7.5
[136]	2016	Shanghai Jiao Tong University	2 µm	BP	3 × 1.2 × 6 mm^3^ Tm (5 at.%): YAG ceramic	11.6	3.12 × 10^3^	3.32
[137]	2017	Belarusian National Technical University	1926	graphene	Tm: KLuW	260	190	4.1
[138]	2018	Qilu University of Technology	1940	SnSe_2_	3 × 3 × 6 mm^3^ Tm (4 at.%): YAP	109.77	1.29 × 10^3^	3.6
[139]	2019	Yantai University	2000.5	Boron nitride	3 × 3 × 8 mm^3^ Tm (5 at.%), Ho (0.3 at.%):YAP	41.7	6.3 × 10^3^	13
[140]	2020	Changchun University of Science and Technology	2006	GaSe	3 × 3 × 5 mm^3^ Tm (2 at.%):YAG	66.8	500	-
[141]	2020	Harbin Engineering University	2 µm	MoS_2_	3 × 3 × 8 mm^3^ Tm (3 at.%): YAP	105.1	916	19.5
[142]	2020	Harbin University of Science and Technology	1988.3	WSe_2_	3 × 3 × 8 mm^3^ Tm (3 at.%): YAP	113.7	392.7	11.3
[143]	2020	Harbin Institute of Technology	2 µm	WS_2_ MoS_2_	3 × 3 × 5 mm^3^ Tm (3 at.%): YAP	34.7 24.0	2.65 × 10^3^ 2.50 × 10^3^	2.9 3.8

Figure 9b,c shows the variations of peak power and pulse width when inserting different saturable absorbers, respectively. Experimental results show that dual passively Q-switched is an effective method to compress the pulse width, increase the peak power, and obtain a stable pulse sequence by comparing it with the placement of saturable absorbers alone. However, as a lidar light source, it is still desirable to obtain the widest possible pulse width.

**Figure 10:**
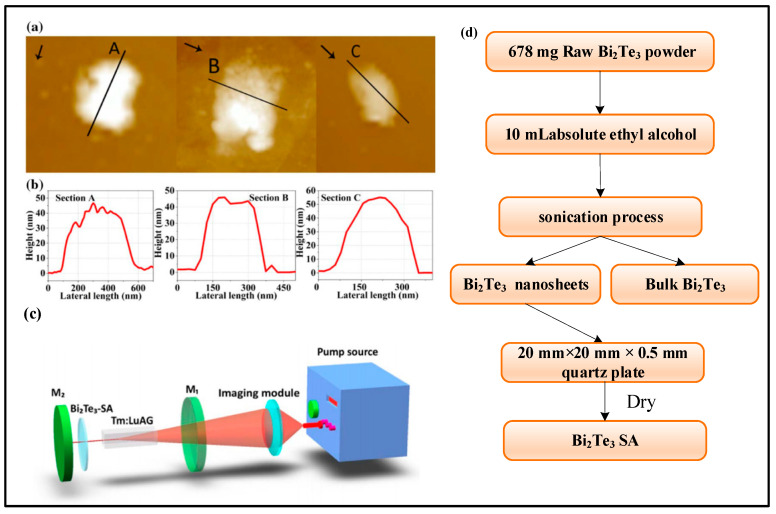
(**a**) Surface morphology of Bi_2_Te_3_ nanosheets. (Three arrows represent the direction of measurement for the height profile); (**b**) The profile height of Bi_2_Te_3_ nanosheets; (**c**) Experimental device diagram; (**d**) Bi_2_Te_3_ nanosheets preparation flow chart [112].

In recent years, manufacturers at home and abroad have further optimized the preparation process of saturable absorbers, such as the sol–gel method, hydrothermal method, chemical vapor deposition method, physical vapor deposition method, and so on. These processes can achieve uniform coating, high-quality growth, and precise control of saturable absorbers, which help them show better modulation characteristics in experiments. The more commonly used method is the liquid phase exfoliation method. The ultrasound-assisted liquid phase exfoliation method is the use of ultrasonic waves to treat the material, which can separate the surface or internal structure of the sample and add appropriate solvents and other reagents to it. Firstly, the sample needs to be placed in a container, and some organic solvents such as surfactants and reducing agents can be added to enhance the separation effect or change the sample properties. Then, under the action of ultrasonic wave, the surface of the sample will be subjected to small impact force and high-speed flow, so that the material is dispersed, broken, or separated. At this time, the required components can be separated from the solution by centrifugation, filtration, or precipitation. It is important to note that power and time should be mastered when performing ultrasonication to avoid unnecessary damage to the sample. In 2017, Bi_2_Te_3_ nanosheets were prepared by ultrasound-assisted liquid phase exfoliation at Shandong University. The preparation process and the surface structure of Bi_2_Te_3_ nanosheets are shown in Figure 10.

**Figure 11:**
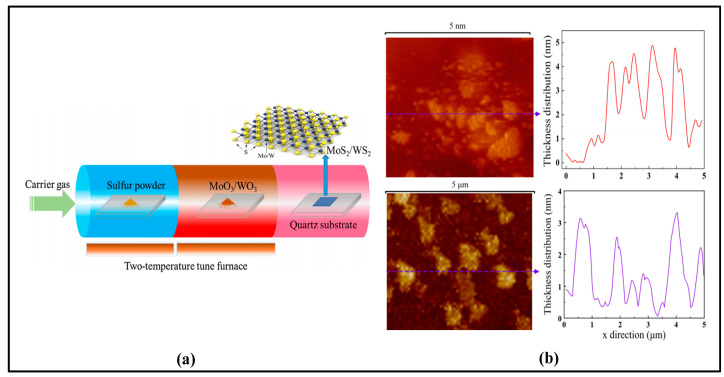
(**a**) The preparation process of WS_2_ and MoS_2_ nanosheets; (**b**) Morphological images and thickness distribution of WS_2_ and MoS_2_. Reprinted with permission from [143] © Elsevier.

Figure 10a shows the surface morphology of the three prepared Bi_2_Te_3_ nanosheets using atomic force microscopy (AFM), and the three arrows indicate the different orientations when measuring the profile height. The thickness of Bi_2_Te_3_ nanosheets can be observed in Figure 10b, which has a very important effect on the modulation depth of saturable absorption as well as on the unsaturation loss. In addition, chemical vapor deposition (CVD) is also a common method for preparing saturable absorbers. Its advantages mainly include high purity, uniformity, controllability, diversity, and low cost. CVD can prepare high-purity saturable absorbers with good homogeneity and stability under high temperatures and high vacuum, and the optical properties of the prepared saturable absorbers can be precisely controlled by adjusting the reaction parameters to meet the needs of lasers in different wavelength ranges. In 2020, WS_2_ and MoS_2_ nanosheets were prepared by chemical vapor deposition at the Harbin Institute of Technology, and the microstructures of these two materials and the optical properties of saturable absorbers were investigated in depth, respectively. The preparation process is shown in Figure 11.

The method of using a saturable absorber directly has the advantages of no external drive, compactness, and flexibility. However, in the experiment, a suitable saturable absorber also needs to be selected to match the wavelength, gain, repetition frequency, and other parameters of the laser and may be affected by the thermal effect, resulting in a decrease in output power or damage to the saturable absorber. It may also produce a multi-pulse output, which affects the output quality and stability, as well as the high price of individual materials and the complexity of the preparation process. These are shortcomings that cannot be ignored, which will also limit the development of 2 µm passively Q-switched technology. From the above table, it can also be seen that the passively Q-switched technology can also obtain a higher repetition frequency, but the single pulse energy is µJ magnitude, which is lower than the mJ magnitude of the actively Q-switched technology. It is not suitable for amplification as a seed light source for coherent Doppler wind lidar. Therefore, most of the current research on passively Q-switched lasers focuses on the study of saturable absorption materials. It is mainly shown that this saturable absorption material can be used as a passively Q-switched device in the 2 µm band.

## 3. Conclusions

In the past decade, the 2 µm solid lidar light source has shown broad application prospects in military, civil, scientific research, and other fields due to its advantages of eye safety, high atmospheric transmittance, and strong anti-interference ability. Therefore, this paper reviews the development status of 2 µm band human eye safety coherent Doppler wind lidar and summarizes and analyzes the 2 µm pulsed single longitudinal mode solid-state laser, 2 µm seed solid-state laser, and 2 µm high power solid-state laser, respectively.

Regarding, the 2 µm seed solid-state laser, the methods of obtaining single longitudinal mode seed light mainly include the Fabry–Perot etalon method, microchip laser method, twisted-mode cavity method, single non-planar ring oscillator method, coupled cavity method, etc. Among them, the most commonly used method is to insert Fabry–Perot etalons into the cavity, because it is simple to operate and has good effects. In 2016, the Harbin Institute of Technology inserted three F-P etalons into the cavity to achieve tunable wavelength output from 2077 nm to 2081 nm, and single longitudinal mode output could be achieved at each wavelength. In the Q-switched laser part of amplifying the single longitudinal mode seed light, it is mainly represented by Changchun University of Science and Technology, the Harbin Institute of Technology, and Shandong University. Among them, Shandong University is in a leading position in passively Q-switched, which proves that a variety of materials have excellent characteristics as passively Q-switched devices. In 2019, it was successfully proved that a dual Q-switched laser using both MoS_2_ and WS_2_ could generate more stable pulse sequences with shorter pulse width and higher peak power. However, as the light source of lidar, actively Q-switched has more advantages with its mJ pulse energy and ns long pulse width output. For the 2 µm pulsed single longitudinal mode laser, injection locking technology can inject continuous single longitudinal mode seed light with narrow linewidth and low power into a high power laser oscillator to realize high energy pulse output, which is the best technology to meet the requirements of a coherent Doppler wind lidar light source.

According to the summary of the 2 µm solid-state lidar light source in the past decade, from the coating process of laser diaphragm, the growth and preparation of new crystals and materials, and the design of resonant cavity, the overall trend of laser performance has been steadily improved compared with that before 2010. In the future, new gain media will continue to be developed for the 2 µm solid-state lidar light source, including by exploring new Ho^3+^-doped laser crystals or ceramic materials to improve the gain coefficient, reduce the thermal lens effect, expand the refractive index modulation range, etc., or by optimizing the pumping method, gain medium structure and cavity design methods, to improve the performance of the 2 µm solid-state laser radar light source, to show more accurate and longer detection capabilities.

The development trend of 2 µm solid-state lasers in the future mainly includes improving materials and designing more optimized resonators and pumping sources to improve power and efficiency. At the same time, on the basis of being widely used in medical treatment, environmental detection, agriculture, remote sensing, and communication, it continues to expand new application fields, such as unmanned air vehicles, intelligent transportation, and the Internet of Things. In addition, it will also develop in a smaller, lighter, and more portable direction to meet the needs of mobile applications and reduce costs and improve reliability.

## Data Availability

Not applicable.
